# Abnormal Echogenicity of the Substantia Nigra, Raphe Nuclei, and Third-Ventricle Width as Markers of Cognitive Impairment in Parkinsonian Disorders: A Cross-Sectional Study

**DOI:** 10.1155/2016/4058580

**Published:** 2016-01-10

**Authors:** Angela E. P. Bouwmans, Albert F. G. Leentjens, Werner H. Mess, Wim E. J. Weber

**Affiliations:** ^1^Department of Neurology, Reinier de Graaf Gasthuis, Reinier de Graafweg 5, 2625 AD Delft, Netherlands; ^2^Department of Psychiatry, Maastricht University Medical Centre, P. Debyelaan 25, 6229 HX Maastricht, Netherlands; ^3^Department of Clinical Neurophysiology, Maastricht University Medical Centre, P. Debyelaan 25, 6229 HX Maastricht, Netherlands; ^4^Department of Neurology, Maastricht University Medical Centre, P. Debyelaan 25, 6229 HX Maastricht, Netherlands

## Abstract

*Background*. Patients with Parkinson's disease (PD) have a high risk of cognitive problems.* Objective*. This study assesses whether abnormal echogenicity of the substantia nigra (SN) and raphe nuclei (RN) and the diameter of third ventricle are markers of cognitive impairment in patients with PD and other forms of parkinsonism.* Methods*. 126 outpatients with early signs of parkinsonism underwent transcranial sonography (TCS). The scales for the outcome of Parkinson's disease cognition (SCOPA-COG) were used as cognitive measure. Definite neurological diagnosis was established after two-year follow-up.* Results*. One-third of the patients with PD and half of those with APS had signs of cognitive impairment. The echogenicity of the SN was not related to cognitive impairment. The diameter of the third ventricle was significantly larger in PD patients with cognitive impairment compared to those without. In patients with APS we found a significantly higher frequency of hypoechogenic RN in patients with cognitive problems.* Conclusions*. Cognitive impairment is already present in a substantial proportion of patients with PD and APS at first referral. In patients with APS the frequency of hypoechogenic RN points to the direction of other pathophysiology with more emphasis on deficits in the serotonergic neurotransmitter system. The larger diameter of the third ventricle in PD patients with cognitive impairment may reflect Alzheimer like brain atrophy, as has been reported in earlier studies.

## 1. Introduction

Although motor symptoms are the characteristic symptoms of Parkinson's disease (PD) and required for the diagnosis, cognitive impairment, especially in executive function [1], also frequently occurs in the early stage of the disease or even as the first symptom [2]. Several studies report that the relative risk of developing dementia in PD is two to six times higher than that of the general population [3, 4]. About half of the patients with PD have mild cognitive impairment (MCI) [5] and are prone to develop dementia [6, 7]. The cumulative incidence of dementia in PD is 66% after 12 years [8] and up to 87% after 20 years [9]. Despite these substantial figures, cognitive dysfunction in PD patients is often not recognized and leads to considerable disease burden [10–14].

Transcranial sonography (TCS) of the mesencephalon has emerged as a possible diagnostic tool for PD, because of its ability to delineate hyperechogenic zones in the substantia nigra (SN) [15, 16]. The echogenicity of the SN has also been mentioned as a marker for the presence of cognitive impairment [17]. Another structure which is normally visible by TCS is the raphe nuclei (RN). If the RN cannot be delineated this has been associated with the presence of depression. It is known that there may be a possible pathologic link between mood and cognition [18–20]. Third-ventricle width can also be estimated and may serve as an indicator of surrounding brain atrophy [21]. This has been thoroughly studied by magnetic resonance scans (MRIs) in patients with neurodegenerative disorders, in particular Alzheimer's disease (AD) [22–24] and also PD [25–27]. In the few studies in which PD patients underwent TCS, the third-ventricle width was significantly larger in patients with cognitive impairment compared to those without cognitive impairment and control subjects [28, 29]. However, in these studies group sizes were small and the disease duration was often very long, so the results may not be representative for patients in an early stage of PD.

The aim of this analysis was to explore the association between cognitive impairment in patients with parkinsonism and echogenic features shown by TCS. We used the dataset of a large prospective cohort study on the diagnostic accuracy of TCS in early parkinsonian patients [30–32]. We hypothesized that hyperechogenic SN, hypoechogenic RN, and a wider diameter of the third ventricle are associated with the presence of cognitive deficits, both in patients with PD as well as in patients with atypical parkinsonian syndromes (APS).

## 2. Methods

### 2.1. Design

This study was a cross-sectional study, nested within a prospective cohort study that aimed to test the diagnostic accuracy of TCS of the SN in patients who were referred to a neurologist by their general practitioner because of recent-onset parkinsonism of unclear origin [30, 32]. The study protocol was published before patient inclusion started [30]. The main finding of the cohort study was that the diagnostic accuracy of the echogenicity of the SN as a diagnostic test for early PD is not sufficient for routine clinical use [32]. The Institutional Review Board (IRB) of Maastricht University Medical Centre approved the study (MEC 05–228, 4 April 2006), which was registered in the ClinicalTrials.gov database as (ITRSCC) NCT00368199.

### 2.2. Patients

**Figure 1:**
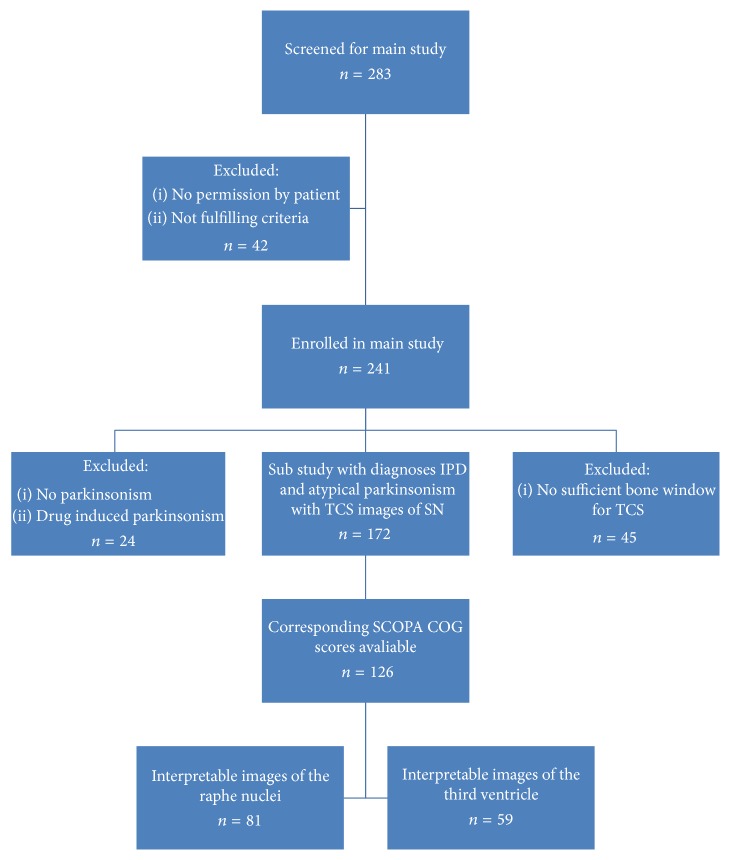


We considered 283 consecutive patients with parkinsonism of unknown origin, who were referred to the neurology outpatient clinic of Maastricht University Medical Centre, Maastricht, and the Orbis Medical Centre, Sittard, Netherlands, for diagnostic work-up. Patients who did not consent or those in whom a definite diagnosis could already be made at the first visit (*n* = 42) were excluded from the study. Hence, 241 patients with parkinsonism of unknown etiology were included. Of these, another 69 were excluded: 24 patients without clear parkinsonian symptoms or with drug-induced parkinsonism, as well as 45 patients without a sufficient bone window necessary to perform an adequate TCS examination (see Figure 1).

Eventually, we were able to obtain interpretable TCS images of the SN in 126 patients. Interpretable images of the RN and third ventricle were available for 81 and 59, respectively, of the 126 since these structures were visualized only in one center or later on in the study.

### 2.3. Measures

After signing informed consent, all subjects underwent a structured interview [30] and a neurological examination [30, 32]. For further information about the interview, we would like to refer to our protocol [30]. These tests were performed by a physician not treating the patient and blinded for information in clinical records. Cognitive function was measured with the scales for the outcome of Parkinson's disease cognition (SCOPA-COG). This scale has a good reliability and validity, both in PD patients and in the general population [33]. The score range is 0 to 43, with scores lower than 22 suggesting clinically relevant cognitive impairment. Depressive symptoms were measured with the observer-rated 17-item Hamilton Depression Rating Scale (HAMD) [34, 35]. Motor symptoms were measured with the Unified Parkinson's Disease Rating Scale (UPDRS) [36].

Within two weeks of inclusion all patients underwent a TCS, at the Department of Clinical Neurophysiology of one of the two hospitals. In the hospital in Sittard only the SN was visualized. In Maastricht University Medical Center, visualization of the RN was included in the TCS protocol from the start of the study. One year later, measurement of the third ventricle was included as well.

TCS was performed using a SONOS 5500 system (Philips, Eindhoven, Netherlands). The examination took place in a darkened room with the patient already lying on the examination table before the investigator entered the room, in order to minimize possible identification of a patient's clinical signs. Patient and investigator were instructed not to discuss symptoms or diagnoses.

TCS was performed bilaterally through the preauricular bone window with a 2–4 MHz phased array transducer. The quality of the bone window was scored as good, moderate, or inferior.

Two different methods were applied for the evaluation of the SN. First, the presence or absence of a clearly visible SN was scored (qualitative method). Second, the SN area was encircled manually and calculated automatically (quantitative method). This was only performed when the hyperechogenicity was located within the anatomical distribution of the SN, meaning that it showed a typical oblique stripe-shaped configuration. Both the right and left SN were measured from both sides.

The RN were identified if they met the criteria of an anatomic structure equally echo-intense to the red nucleus and localized in the transverse plane of the midbrain extending uninterrupted from anterior to posterior direction. Echogenicity of the RN was rated using a visual scoring system resulting in a semiquantitative assessment. Hence, if the RN appeared as an uninterrupted relatively echo-intense structure on TCS, it was scored as hyperechogenic. We scored the RN as hypoechogenic if it was not detectable at all or if it was interrupted. The patient was scanned from both sides because of the fact that the bone window can vary in quality of visualization of the RN from right to left. We used the best possible result, meaning when the RN was absent from one side but visible from the other side, it was scored as hyperechogenic.

The transverse diameter of the third ventricle was measured from both sides on a standardized diencephalic examination plane.

Two years after inclusion, patients were reexamined by two movement disorder neurologist specialists to obtain a definite clinical diagnosis, using the official diagnostic criteria for Parkinson's disease [35] as well as for several atypical parkinsonian disorders, including multisystem atrophy (MSA), progressive supranuclear palsy (PSP), Lewy Body Disease (LBD), corticobasal degeneration (CBD) [37–41], essential tremor (ET) [42, 43], and vascular parkinsonism (VP) [44] which served as gold standard for our study [30].

### 2.4. Statistics

SPSS 21.0 for Windows was used for the statistical analysis. Comparing categorical variables was done by chi-square test. The two-sample *t*-test was used for comparing continuous variables.

For further analyses regarding the means between groups with different diagnoses, we performed an analysis of variance (ANOVA). To analyse the influence of other variables, partial correlation analysis was used. *p* values of <0.05 were considered significant.

## 3. Results

### 3.1. Patient Characteristics

At follow-up, seventy-two (57%) patients were diagnosed with PD. Patients with APS included 7 patients with MSA, 4 with PSP, 4 having LBD, and 3 patients with CBD. Eighteen (14%) patients had VP and 18 (14%) were diagnosed with ET.

At inclusion, 81% of the patients did not use any medication which could have an influence on both the motor as the cognitive performance such as an antidepressant or antiepileptic medication.

The subgroups differed significantly on the total UPDRS score with patients with APS having higher UPDRS scores compared to those with PD (Table 1). PD patients tended to be younger and having higher scores on the SCOPA-COG (*p* = 0.05). However no significant correlation was found between the SCOPA-COG score and UPDRS total score (Pearson correlation coefficient −0.204, *p* = 0.127), nor between the SCOPA-COG score and age of the patient (Pearson correlation coefficient −0.145, *p* = 0.277), in the total study group, as well as in the two subsamples separately.

Fifty-five (44%) subjects of the total group had a SCOPA-COG < 22, indicating the presence of cognitive impairment. Of the 72 PD patients, 27 (37.5%) had a SCOPA-COG < 22 compared to 28 (52%) of the APS patients. The mean score on the HAMD was 5.05 (SD = 5.40) with a range of 0 up to 28.

### 3.2. Echogenicity

**Figure 2:**
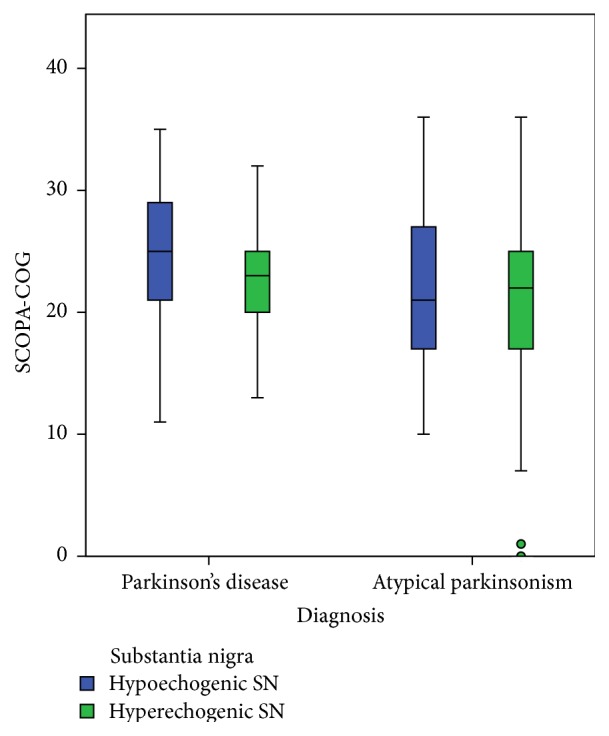
Boxplot of the range of scores on the SCOPA-COG scores compared to the echogenicity of the substantia nigra.

**Figure 3:**
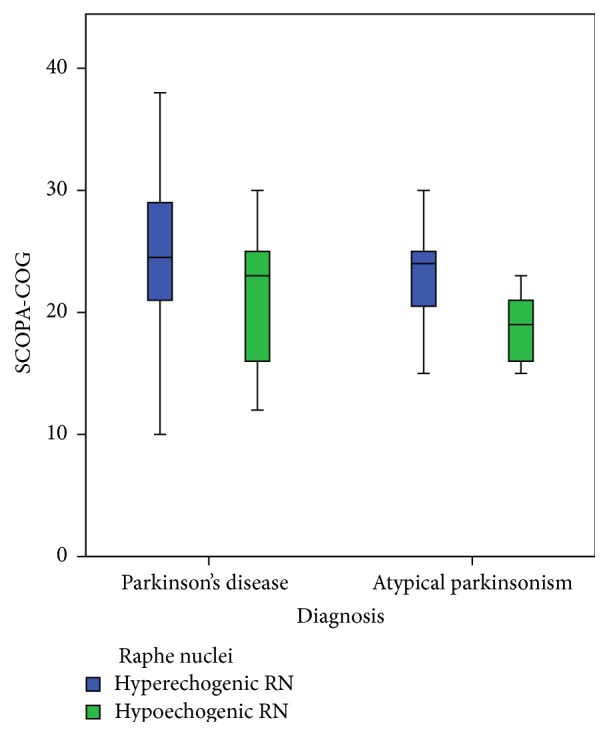
Boxplot of the range of scores on the SCOPA-COG scores compared to the echogenicity of the raphe nuclei.

**Figure 4:**
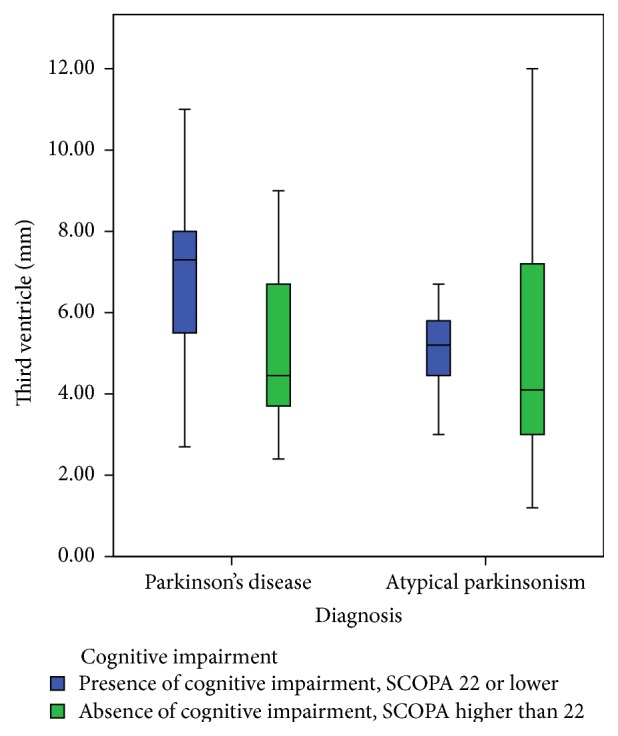
Boxplot of the range of scores on the SCOPA-COG scores compared to the diameter of the third ventricle divided in subgroups with and without cognitive impairment.

The percentage of abnormal SN or RN echogenicity, as well as third-ventricle width, did not differ between the PD patients and the patients with APS (see Table 1). The echogenicity of the SN was not related to the presence or absence of cognitive impairment, neither in PD nor in APS (Figure 2). However, in patients with APS, but not in PD patients, we found a significantly higher frequency of hypoechogenicity of the RN in patients with cognitive impairment compared to those without (Table 2, Figure 3). No significant differences between the mean of Hamilton scores had been seen between those with a hyperechogenic RN and those with a hypoechogenic RN: 5.30 (SD = 6.39) and 4.93 (SD = 5.24), respectively; *t* = 0.203, *p* = 0.840. The diameter of the third ventricle was significantly larger in PD patients with cognitive impairment compared to those PD patients without. There was no difference in third-ventricle width in APS patients with and without cognitive impairment (Table 2, Figure 4).

## 4. Discussion

### 4.1. Statement of Principal Findings

About one-third of the patients with PD and about half of the patients with APS had cognitive impairment. The echogenicity of the SN did not indicate the presence or absence of cognitive impairment, neither in PD nor in APS. In patients with APS we found a significantly higher frequency of hypoechogenic RN in patients with cognitive impairment compared to the patients without cognitive impairment. Furthermore, we found a larger diameter of the third ventricle in PD patients with cognitive impairment compared to the PD patients without cognitive impairment.

### 4.2. Strengths and Weaknesses of the Study

The major limitation of our study is that it is a secondary analysis of a study that was not powered on the presence of cognitive impairment. Because of that, our results must be seen as exploratory and interpreted with caution. However, the prevalence of cognitive impairment in our study is comparable to that in other studies [7, 45]. Because of new developments in TCS after start inclusion, we decided at a later point to also investigate the RN and the third ventricle resulting in a lower number of these echo features.

The important strengths of our study are that, compared to other studies, sample size is fairly large regarding the amount of visualized SN and the fact that we included newly referred patients with early signs of parkinsonism that are not yet (extensively) treated pharmacologically. This patient group is clinically the most relevant, because in daily practice you want to know the right diagnosis in the earliest stage of the disease.

### 4.3. Interpretation of Results

A study performed in PD patients with and without cognitive impairment and in patients with LBD reported a hyperechogenic SN in almost all patients [46]. This does not seem to reflect Lewy body pathology, however, since in other forms of parkinsonism, where Lewy bodies usually are not found such as in CBD and patients with parkin-related parkinsonism, hyperechogenic SN has also been reported [47, 48]. The hyperechogenicity may reflect early alteration of dopaminergic cells resulting from neurodegeneration [48] caused by increased abnormal-bound iron [49]. However, a hyperechogenic SN can also be detected in some other nonneurodegenerative diseases [50] and even in normal aging [51, 52]. Hence, there is uncertainty about the pathophysiological substrate of an increased echogenicity of the SN as well as the impact on a given patient. In our study we did not find any difference in hyperechogenicity of the SN in PD or APS patients with and without cognitive impairment. This may be an indication that the SN plays a less pivotal role in cognitive functioning than previously thought. Moreover, morphological changes in a structure do not necessarily result in functional changes because compensation mechanisms of other neuronal networks may come into action.

Hypoechogenicity of the RN has been described as a indicator for the presence of depression in various diseases [53–55]. It has been suggested that depression and cognitive impairment share at least in part the same underlying pathology [18–20]. In our study we found a higher prevalence of a hypoechogenicity of the RN in APS patients with cognitive impairment compared to those without cognitive impairment. Hypoechogenicity of the RN may indicate reduction of serotonin receptors [56] which may affect cognitive function [57, 58].

The finding of an association between cognitive impairment and a larger diameter of the third ventricle in our studies confirms earlier studies in PD, LBD, and AD [29, 46, 59]. Increased size of the ventricles is a marker for cognitive dysfunction in PD [29]. Increased ventricular size could be a nonspecific sign of atrophy in the surrounding structures such as the brainstem nuclei, due to progressive neurodegeneration in AD [21]. The rate of change of ventricular size seems to correlate with an increase in senile plaques and neurofibrillary tangles [59].

How can we explain that in two different diagnostic groups (PD and APS) different echo features were related to the presence of cognitive impairment? This is likely due to the different underlying pathophysiology. Earlier studies have described a pattern of brain atrophy which is similar in AD and in PD [60–62]. The characteristic cognitive dysfunction in AD (affecting memory) and PD (affecting executive function) is different. It may be that cognitive impairment in PD may be segregated into two different types [63]: one due to dysfunction of the dopaminergic frontostriatal networks leading to executive problems and one primarily due to posterior cortically deficits resulting from AD-pathology [64–66]. This latter type may be related to abnormal dilatation of the third ventricle. Indeed amyloid pathology, which is the principal pathology in AD, has also been reported to play a role in cognitive dysfunction in PD and LBD [67, 68].

With regard to hypoechogenicity of the RN in APS, because these patients have a worse prognosis and faster disease progression than PD, the emphasis of the pathophysiology may lay more on neurotransmitter level than on decline through atrophy. There also seems to be a correlation between the SCOPA-COG score and the score on the UPDRS which may explain the higher frequency of cognitive problems in APS compared to PD.

### 4.4. Unanswered Questions and Future Research

The exact pathogenesis of cognitive impairment in PD is still unknown. Studies have indicated that Lewy-type pathology [69, 70] and associated Alzheimer type histological changes [2], together with neurotransmitter deficits [71–73], especially the reduced cholinergic activity, are involved. However, to which extent and in which stage of the disease each factor has its contribution remains unclear.

## 5. Conclusion

The echogenicity of the SN was not related to cognitive impairment, neither in PD nor in APS. In patients with APS the frequency of hypoechogenic RN points to the direction of other pathophysiology with more emphasis on deficits in the serotonergic neurotransmitter system. The larger diameter of the third ventricle in PD patients with cognitive impairment compared to the PD patients without supports earlier findings that the width of the third ventricle may reflect Alzheimer like brain atrophy. Differences shown by TCS indicate that various mechanisms and pathways are involved in the pathophysiology of cognitive impairment in PD and APS.

## Figures and Tables

**Table 1:** Patient characteristics, divided into group of diagnosis.

	Parkinson's disease (*n* = 72)	Atypical parkinsonism (*n* = 54)	*p* value
Age years (SD)	68.54 (9.27)	71.85 (9.31)	*0.05*
Men (%)	50 (70)	43 (80)	0.20
Disease duration months mean (SD)	29.08 (46.78)	40.39 (41.69)	0.16
UPDRS total score mean (SD)	24.03 (10.98)	30.26 (17.24)	***0.02***
UPDRS motor score mean (SD)	13.10 (6.00)	15.63 (8.52)	0.07
SCOPA-COG mean (SD)	23.83 (5.93**)**	21.41 (7.85)	*0.05*
Presence of cognitive impairment %	37.50	51.85	0.11
Hyperechogenic substantia nigra %	41.28	46.30	0.50
Hypoechogenic raphe nuclei %	21.74	20.00	0.85
Third-ventricle width mm (SD)	5.78 (2.28)	5.31 (2.38)	0.45

SCOPA-COG = scales for the outcome of Parkinson's disease cognition, UPDRS = Unified Parkinson's Disease Rating Scale.

**Table 2:** Patient characteristics, divided into presence or absence of cognitive impairment.

	PD absence of cognitive impairment (*n* = 45)	PD presence of cognitive impairment (*n* = 27)	*p* value	APS absence of cognitive impairment (*n* = 26)	APS presence of cognitive impairment (*n* = 28)	*p* value
Mean age years (SD)	67.27 (10.10)	70.67 (7.36)	0.13	68.15 (10.53)	75.29 (6.49)	*0.05*
Disease duration months mean (SD)	27.29 (53.75)	32.07 (32.87)	0.68	51.92 (50.28)	29.68 (28.78)	*0.05*
UPDRS total score mean (SD)	22.31 (10.14)	27.00 (11.90)	0.83	25.27 (13.77)	35.07 (19.05)	**0.04**
UPDRS motor score mean (SD)	12.56 (5.55)	14.04 (6.73)	0.32	13.31 (6.75)	17.79 (9.50)	*0.05*
SCOPA-COG mean (SD)	27.40 (3.70)	17.89 (3.79)	**0.00**	27.54 (4.26)	15.71 (5.87)	**0.00**
Hyperechogenic substantia nigra %	35.56	48.15	0.29	46.15	46.43	0.98
Hypoechogenic raphe nuclei %	20.69	23.53	0.82	5.56	35.29	**0.03**
Third-ventricle width mm (SD)	5.05 (1.84)	6.92 (2.49)	**0.02**	5.27 (3.02)	5.38 (1.44)	0.91

PD = Parkinson's disease, APS = atypical parkinsonism.

## References

[1] Dubois B., Pillon B. (1997). Cognitive deficits in Parkinson's disease. *Journal of Neurology*.

[2] Caballol N., Martí M. J., Tolosa E. (2007). Cognitive dysfunction and dementia in Parkinson disease. *Movement Disorders*.

[3] Emre M. (2003). Dementia associated with Parkinson's disease. *Lancet Neurology*.

[4] Aarsland D., Kurz M. W. (2010). The epidemiology of dementia associated with Parkinson's disease. *Brain Pathology*.

[5] Caviness J. N., Driver-Dunckley E., Connor D. J. (2007). Defining mild cognitive impairment in Parkinson's disease. *Movement Disorders*.

[6] Williams-Gray C. H., Foltynie T., Brayne C. E. G., Robbins T. W., Barker R. A. (2007). Evolution of cognitive dysfunction in an incident Parkinson's disease cohort. *Brain*.

[7] Janvin C., Aarsland D., Larsen J. P., Hugdahl K. (2003). Neuropsychological profile of patients with Parkinson's disease without dementia. *Dementia and Geriatric Cognitive Disorders*.

[8] Buter T. C., van den Hout A., Matthews F. E., Larsen J. P., Brayne C., Aarsland D. (2008). Dementia and survival in Parkinson disease: a 12-year population study. *Neurology*.

[9] Hely M. A., Reid W. G. J., Adena M. A., Halliday G. M., Morris J. G. L. (2008). The Sydney Multicenter Study of Parkinson's disease: the inevitability of dementia at 20 years. *Movement Disorders*.

[10] Riedel O., Klotsche J., Spottke A. (2010). Frequency of dementia, depression, and other neuropsychiatric symptoms in 1,449 outpatients with Parkinson's disease. *Journal of Neurology*.

[11] Duncan G. W., Khoo T. K., Yarnall A. J. (2014). Health-related quality of life in early Parkinson's disease: the impact of nonmotor symptoms. *Movement Disorders*.

[12] Martinez-Martin P. (2011). The importance of non-motor disturbances to quality of life in Parkinson's disease. *Journal of the Neurological Sciences*.

[13] Hughes T. A., Ross H. F., Mindham R. H. S., Spokes E. G. S. (2004). Mortality in Parkinson's disease and its association with dementia and depression. *Acta Neurologica Scandinavica*.

[14] Morley D., Dummett S., Peters M. (2012). Factors influencing quality of life in caregivers of people with Parkinson's disease and implications for clinical guidelines. *Parkinson's Disease*.

[15] Berg D., Gaenslen A. (2010). Place value of transcranial sonography in early diagnosis of Parkinson's disease. *Neurodegenerative Diseases*.

[16] Bouwmans A. E. P., Vlaar A. M. M., Srulijes K., Mess W. H., Weber W. E. J. (2010). Transcranial sonography for the discrimination of idiopathic Parkinson's disease from the atypical Parkinsonian syndromes. *International Review of Neurobiology*.

[17] Liepelt I., Wendt A., Schweitzer K. J. (2008). Substantia nigra hyperechogenicity assessed by transcranial sonography is related to neuropsychological impairment in the elderly population. *Journal of Neural Transmission*.

[18] Panza F., Frisardi V., Capurso C. (2010). Late-life depression, mild cognitive impairment, and dementia: possible continuum?. *The American Journal of Geriatric Psychiatry*.

[19] Monastero R., Mangialasche F., Camarda C., Ercolani S., Camarda R. (2009). A systematic review of neuropsychiatric symptoms in mild cognitive impairment. *Journal of Alzheimer's Disease*.

[20] Hermida A. P., McDonald W. M., Steenland K., Levey A. (2012). The association between late-life depression, mild cognitive impairment and dementia: is inflammation the missing link?. *Expert Review of Neurotherapeutics*.

[21] Wollenweber F. A., Schomburg R., Probst M. (2011). Width of the third ventricle assessed by transcranial sonography can monitor brain atrophy in a time- and cost-effective manner—results from a longitudinal study on 500 subjects. *Psychiatry Research: Neuroimaging*.

[22] Slansky I., Herholz K., Pietrzyk U. (1995). Cognitive impairment in Alzheimer's disease correlates with ventricular width and atrophy-corrected cortical glucose metabolism. *Neuroradiology*.

[23] Murphy D. G. M., DeCarli C. D., Daly E. (1993). Volumetric magnetic resonance imaging in men with dementia of the Alzheimer type: correlations with disease severity. *Biological Psychiatry*.

[24] Apostolova L. G., Green A. E., Babakchanian S. (2012). Hippocampal atrophy and ventricular enlargement in normal aging, Mild Cognitive Impairment (MCI), and Alzheimer disease. *Alzheimer Disease & Associated Disorders*.

[25] Ibarretxe-Bilbao N., Junque C., Marti M. J., Tolosa E. (2011). Brain structural MRI correlates of cognitive dysfunctions in Parkinson's disease. *Journal of the Neurological Sciences*.

[26] Melzer T. R., Watts R., MacAskill M. R. (2012). Grey matter atrophy in cognitively impaired Parkinson's disease. *Journal of Neurology, Neurosurgery and Psychiatry*.

[27] Burton E. J., McKeith I. G., Burn D. J., Williams E. D., O'Brien J. T. (2004). Cerebral atrophy in Parkinson's disease with and without dementia: a comparison with Alzheimer's disease, dementia with Lewy bodies and controls. *Brain*.

[28] Camicioli R., Sabino J., Gee M. (2011). Ventricular dilatation and brain atrophy in patients with Parkinson's disease with incipient dementia. *Movement Disorders*.

[29] Dalaker T. O., Zivadinov R., Ramasamy D. P. (2011). Ventricular enlargement and mild cognitive impairment in early Parkinson's disease. *Movement Disorders*.

[30] Vlaar A. M. M., Bouwmans A. E. P., van Kroonenburgh M. J. P. G. (2007). Protocol of a prospective study on the diagnostic value of transcranial duplex scanning of the substantia nigra in patients with parkinsonian symptoms. *BMC Neurology*.

[31] Vlaar A. M. M., de Nijs T., Van Kroonenburgh M. J. P. G. (2008). The predictive value of transcranial duplex sonography for the clinical diagnosis in undiagnosed parkinsonian syndromes: comparison with SPECT scans. *BMC Neurology*.

[32] Bouwmans A. E. P., Vlaar A. M. M., Mess W. H., Kessels A., Weber W. E. J. (2013). Specificity and sensitivity of transcranial sonography of the substantia nigra in the diagnosis of Parkinson's disease: prospective cohort study in 196 patients. *BMJ Open*.

[33] Dubois B., Burn D., Goetz C. (2007). Diagnostic procedures for Parkinson's disease dementia: recommendations from the movement disorder society task force. *Movement Disorders*.

[34] Schrag A., Barone P., Brown R. G. (2007). Depression rating scales in Parkinson's disease: critique and recommendations. *Movement Disorders*.

[35] Leentjens A. F. G., Verhey F. R. J., Lousberg R., Spitsbergen H., Wilmink F. W. (2000). The validity of the Hamilton and Montgomery-Asberg depression rating scales as screening and diagnostic tools for depression in Parkinson's disease. *International Journal of Geriatric Psychiatry*.

[36] Movement Disorder Society Task Force on Rating Scales for Parkinson's Disease (2003). The Unified Parkinson's Disease Rating Scale (UPDRS): status and recommendations. *Movement Disorders*.

[37] Hughes A. J., Daniel S. E., Kilford L., Lees A. J. (1992). Accuracy of clinical diagnosis of idiopathic Parkinson's disease: a clinico-pathological study of 100 cases. *Journal of Neurology Neurosurgery and Psychiatry*.

[38] Gilman S., Wenning G. K., Low P. A. (2008). Second consensus statement on the diagnosis of multiple system atrophy. *Neurology*.

[39] Litvan I., Agid Y., Calne D. (1996). Clinical research criteria for the diagnosis of progressive supranuclear palsy (Steele-Richardson-Olszewski syndrome): report of the NINDS-SPSP International Workshop. *Neurology*.

[40] McKeith I. G., Dickson D. W., Lowe J. (2005). Diagnosis and management of dementia with Lewy bodies: third report of the DLB Consortium. *Neurology*.

[41] Litvan I., Grimes D. A., Lang A. E. (1999). Clinical features differentiating patients with postmortem confirmed progressive supranuclear palsy and corticobasal degeneration. *Journal of Neurology*.

[42] Deuschl G., Bain P., Brin M. (1998). Consensus statement of the Movement Disorder Society on Tremor. Ad Hoc Scientific Committee. *Movement Disorders*.

[43] Gironell A., Kulisevsky J. (2009). Diagnosis and management of essential tremor and dystonic tremor. *Therapeutic Advances in Neurological Disorders*.

[44] Glass P. G., Lees A. J., Bacellar A., Zijlmans J., Katzenschlager R., Silveira-Moriyama L. (2012). The clinical features of pathologically confirmed vascular Parkinsonism. *Journal of Neurology, Neurosurgery and Psychiatry*.

[45] Foltynie T., Brayne C. E. G., Robbins T. W., Barker R. A. (2004). The cognitive ability of an incident cohort of Parkinson's patients in the UK. The CamPaIGN Study. *Brain*.

[46] Walter U., Dressler D., Wolters A., Wittstock M., Greim B., Benecke R. (2006). Sonographic discrimination of dementia with Lewy bodies and Parkinson's disease with dementia. *Journal of Neurology*.

[47] Walter U., Dressler D., Wolters A., Probst T., Grossmann A., Benecke R. (2004). Sonographic discrimination of corticobasal degeneration vs progressive supranuclear palsy. *Neurology*.

[48] Walter U., Klein C., Hilker R., Benecke R., Pramstaller P. P., Dressler D. (2004). Brain parenchyma sonography detects preclinical parkinsonism. *Movement Disorders*.

[49] Berg D., Roggendorf W., Schröder U. (2002). Echogenicity of the substantia nigra: association with increased iron content and marker for susceptibility to nigrostriatal injury. *Archives of Neurology*.

[50] Walter U. (2011). Substantia nigra hyperechogenicity is a risk marker of Parkinson's disease: no. *Journal of Neural Transmission*.

[51] Hagenah J., König I. R., Sperner J. (2010). Life-long increase of substantia nigra hyperechogenicity in transcranial sonography. *NeuroImage*.

[52] Behnke S., Double K. L., Duma S. (2007). Substantia nigra echomorphology in the healthy very old: correlation with motor slowing. *NeuroImage*.

[53] Becker G., Struck M., Bogdahn U., Becker T. (1994). Echogenicity of the brainstem raphe in patients with major depression. *Psychiatry Research: Neuroimaging*.

[54] Becker G., Becker T., Struck M. (1995). Reduced echogenicity of brainstem raphe specific to unipolar depression: a transcranial color-coded real-time sonography study. *Biological Psychiatry*.

[55] Walter U., Prudente-Morrissey L., Herpertz S. C., Benecke R., Hoeppner J. (2007). Relationship of brainstem raphe echogenicity and clinical findings in depressive states. *Psychiatry Research—Neuroimaging*.

[56] Andrade R., Haj-Dahmane S. (2013). Serotonin neuron diversity in the dorsal raphe. *ACS Chemical Neuroscience*.

[57] Gasbarri A., Pompili A. (2014). Serotonergic 5-HT7 receptors and cognition. *Reviews in the Neurosciences*.

[58] Terry A. V., Buccafusco J. J., Wilson C. (2008). Cognitive dysfunction in neuropsychiatric disorders: selected serotonin receptor subtypes as therapeutic targets. *Behavioural Brain Research*.

[59] Silbert L. C., Quinn J. F., Moore M. M. (2003). Changes in premorbid brain volume predict Alzheimer's disease pathology. *Neurology*.

[60] Weintraub D., Dietz N., Duda J. E. (2012). Alzheimer's disease pattern of brain atrophy predicts cognitive decline in Parkinson's disease. *Brain*.

[61] Pavese N. (2012). PET studies in Parkinson's disease motor and cognitive dysfunction. *Parkinsonism and Related Disorders*.

[62] Jokinen P., Scheinin N., Aalto S. (2010). *[*
^11^C*]*PIB-, *[*
^18^F*]*FDG-PET and MRI imaging in patients with Parkinson's disease with and without dementia. *Parkinsonism & Related Disorders*.

[63] Goldman J. G., Williams-Gray C., Barker R. A., Duda J. E., Galvin J. E. (2014). The spectrum of cognitive impairment in Lewy body diseases. *Movement Disorders*.

[64] Williams-Gray C. H., Mason S. L., Evans J. R. (2013). The CamPaIGN study of Parkinson's disease: 10-year outlook in an incident population-based cohort. *Journal of Neurology, Neurosurgery and Psychiatry*.

[65] Muslimović D., Schmand B., Speelman J. D., de Haan R. J. (2007). Course of cognitive decline in Parkinson's disease: a meta-analysis. *Journal of the International Neuropsychological Society*.

[66] Garcia-Garcia D., Clavero P., Salas C. G. (2012). Posterior parietooccipital hypometabolism may differentiate mild cognitive impairment from dementia in Parkinson's disease. *European Journal of Nuclear Medicine and Molecular Imaging*.

[67] Donaghy P., Thomas A. J., O'Brien J. T. (2015). Amyloid PET imaging in Lewy body disorders. *American Journal of Geriatric Psychiatry*.

[68] Buongiorno M., Compta Y., Martí M. J. (2011). Amyloid-*β* and *τ* biomarkers in Parkinson's disease-dementia. *Journal of the Neurological Sciences*.

[69] Apaydin H., Ahlskog J. E., Parisi J. E., Boeve B. F., Dickson D. W. (2002). Parkinson disease neuropathology: later-developing dementia and loss of the levodopa response. *Archives of Neurology*.

[70] Duda J. E. (2004). Pathology and neurotransmitter abnormalities of dementia with Lewy bodies. *Dementia and Geriatric Cognitive Disorders*.

[71] Hilker R., Thomas A. V., Klein J. C. (2005). Dementia in Parkinson disease: functional imaging of cholinergic and dopaminergic pathways. *Neurology*.

[72] Huang C., Mattis P., Perrine K., Brown N., Dhawan V., Eidelberg D. (2008). Metabolic abnormalities associated with mild cognitive impairment in Parkinson disease. *Neurology*.

[73] Meyer P. M., Strecker K., Kendziorra K. (2009). Reduced *α*4*β*2^*^-nicotinic acetylcholine receptor binding and its relationship to mild cognitive and depressive symptoms in Parkinson disease. *Archives of General Psychiatry*.

